# Pathological aspects of the assessment of head and neck cancers: United Kingdom National Multidisciplinary Guidelines

**DOI:** 10.1017/S0022215116000451

**Published:** 2016-05

**Authors:** T R Helliwell, T E Giles

**Affiliations:** 1 Department of Cellular Pathology, Liverpool Clinical Laboratories, University of Liverpool, UK; 2 Department of Cellular Pathology, Liverpool Clinical Laboratories, Liverpool, UK

## Abstract

This is the official guideline endorsed by the specialty associations involved in the care of head and neck cancer patients in the UK. It introduces the current best practice in histopathology and cytopathology as it pertains to head and neck and thyroid cancers.

**Recommendations:**

• Accurate diagnosis of the type of malignancy is a key component of effective management. (R)

• Surgeons and oncologists should understand the scope and limitations of cellular pathology in order to inform multidisciplinary discussions. (R)

• A clinically suspected diagnosis of malignancy should be confirmed by biopsy or cytology before operation. (R)

• Cytopathological diagnoses should be discussed with surgeons and radiologists to maximise the information gained from each modality of investigation. (R)

• Pathological investigations are the basis for accurate cancer staging and stratification of clinical outcomes. (R)

## Introduction

This paper is an overview of the use of laboratory investigations and focuses on the important elements of cancer pathology reports that clinicians should use when discussing the implications of a diagnosis and management options with patients and with colleagues in a multidisciplinary setting. The recommendations for pathology practice are based on published evidence; key references are provided in the World Health Organisation (WHO) Classification of Tumours^1^ and in the series of Histopathology Datasets published by the Royal College of Pathologists.^2^^,^^3^ Pathologists have critically important roles in confirming or excluding specific diseases on the basis of cytology or diagnostic biopsy, in assessing the adequacy of treatment, recognising key predictive and prognostic factors, and in contributing evidence-based criteria for the appropriate stratification of clinical outcomes.

## Use of cellular pathology services

### Frozen section

Patient management should be guided primarily by pre-operative biopsy and/or fine needle aspiration (FNA) cytology. Intra-operative frozen sections have a limited role and are appropriately used for the assessment of surgical excision margins when there is clinical doubt as to adequacy.^4^ Frozen sections are occasionally used to confirm the diagnosis of branchial cleft cysts in older people, of papillary, medullary or anaplastic thyroid carcinomas^5^ or to identify lymph node involvement in thyroid cancers; they should not be used to differentiate follicular thyroid carcinoma from adenoma or follicular variant papillary carcinoma. It should be appreciated that the quality of frozen sections is not as good as paraffin sections and that important information may be missed or destroyed through inappropriate use of frozen sections, particularly if small pieces of tissue are submitted for examination.

### Definitive operative specimen

Specimens should be submitted in an adequate amount of 10 per cent neutral buffered formalin (at least three times the volume of the specimen) unless there is prior agreement with the laboratory.^2^ The site and nature of each specimen should be clearly described on the request form and should be appropriately orientated. The form must include the clinical indication for the operation, the duration of signs and symptoms, pre-operative radiotherapy (RT) or chemotherapy, and details of previous biopsies or cytological investigations, and relevant biochemistry (particularly for thyroid diseases).

### Lymph node specimens

The site of origin of lymph nodes should be recorded, and formal neck dissections should clearly state which nodal groups are included and should be clearly orientated, preferably with a diagram.^6^ The optimal handling of biopsies for suspected lymphoma should be discussed with the laboratory; it is often useful to collect fresh tissue in a transport medium for possible cytogenetic and molecular studies.

The predictive value of sentinel node biopsy is now recognised and is becoming established practice, particularly for the early-stage oral carcinoma.^7^ The pathological assessment of sentinel nodes is highly demanding of laboratory time and expertise, involving multiple sections and immunocytochemistry.^8^ This should only be undertaken if appropriately resourced.

### Resection specimens including bone

When cancer resection specimens contain bone, it is often possible to obtain a preliminary report on the soft tissue components of the specimen while the bone is decalcified before processing the tissues to assess the extent of bone invasion and bony margins. Decalcification may take several days or weeks depending on the density of the bone.

### Immunocytochemistry and molecular pathology

Immunocytochemistry plays an important role in the correct diagnosis of primary head and neck cancers, particularly for the less common entities. The prognostic value of assessing oropharyngeal carcinomas for evidence of human papilloma virus infection (HPV) is established, with current guidance recommending a combination of immunocytochemistry for p16 protein overexpression and in situ hybridisation for high-risk HPV DNA. Morphologically similar poorly differentiated carcinomas arising in the oropharynx and nasopharynx, and their nodal metastases may be distinguished by the presence of HPV and Epstein–Barr virus DNA, respectively.

In patients with metastatic malignancy in cervical lymph nodes without evidence of primary disease, the morphological features of the metastatic tumour may be useful, e.g. thyroid and salivary neoplasms. Immunocytochemical investigation of FNA or biopsy material does not reliably distinguish between primary sites of squamous cell carcinomas (SCCs) but may be helpful in identifying adenocarcinomas arising in the gastrointestinal tract, lungs or prostate. Clinicians should note that immunocytochemical markers are very rarely specific for particular tissues and that opinions on likely primary sites are based on the assessment of a panel of different markers and the balance of probabilities. Clinical features, such as the pattern of nodal disease, and imaging studies should be incorporated into the multidisciplinary assessment of these patients. Molecular genetic profiling of head and neck cancers is not currently recommended outside the research setting.^2^^,^^9^^,^^10^

## Multidisciplinary team working

Cellular pathologists are core members of cancer MDTs and are essential to the provision of a successful service. The MDT should have a risk-based approach to developing its policy on pathology review, particularly for patients who have had diagnostic biopsies in other hospitals. Pathological review is essential for thyroid cancers and is good practice for other situations.

## Malignancies of the upper aerodigestive tract

### Squamous cell carcinoma

The initial diagnosis may be obvious clinically on the basis of an irregularly infiltrating mass with ulceration, but should always be confirmed by biopsy as some inflammatory diseases, e.g. tuberculosis and sarcoidosis, can mimic carcinomas clinically and other mucosal malignancies, e.g. lymphoma, may require consideration of other treatment options. Practical problems that may preclude definitive diagnosis on diagnostic biopsies include poor orientation, necrotic or inflammatory debris, small samples containing few cells and crush artefact. The edges of laser resection specimens often show thermal artefacts, making detailed interpretation impossible. Patients who have been treated with RT and/or chemotherapy may have biopsies or resections to assess any residual or recurrent disease at primary or nodal sites. Extensive scarring, radiation-associated nuclear atypia and loss of the normal anatomical landmarks may make assessment of these specimens difficult. A good chemotherapeutic response may leave a mass of necrotic tissue containing degenerate keratinocytes; viable carcinoma may not be identified even after extensive histological sampling.

#### Morphological variants of SCC

Some variants of SCC are associated with particular difficulties in diagnosis and clinical assessment but should be managed, stage for stage, in line with classical carcinomas.

Papillary SCC is typified by an exophytic growth pattern with fronds of fibrovascular tissue covered by squamous epithelium showing in situ carcinoma; areas of invasive carcinoma are often small and limited in extent. Diagnostic biopsies may show only in situ carcinoma despite a bulky tumour. The prognosis is relatively good due to the limited invasive component.

Verrucous SCC has an exophytic growth and is formed by extremely well-differentiated squamous epithelium with minimal atypia and abundant surface keratin. Diagnostic biopsies may not show invasion and the minimal cellular atypia makes pathologists reluctant to diagnose malignancy. Repeated biopsies and appreciation of the discrepancy between a clinically obvious carcinoma and minimal microscopic atypia are often needed to make a diagnosis of carcinoma.

Spindle cell carcinomas typically present as polypoid tumours with an ulcerated surface and are formed by sheets of atypical spindle cells, often raising the possibility of sarcoma. Sarcomas of mucosal origin are extremely rare in adults, but a definitive diagnosis of spindle cell carcinoma may only be possible on resection specimens when small areas of in situ or more typical invasive carcinoma are identified. Immunohistochemistry only identifies squamous epithelial differentiation in about 60–70 per cent of cases.

Oropharyngeal SCCs are usually related to high-risk HPV infection. Typical HPV-associated carcinomas are non-keratinising (basaloid) carcinomas, but may be of any histological type.

#### Information that should be provided in histopathology reports

The information available from diagnostic biopsies is limited but should normally include whether any carcinoma is invasive or in situ and, for invasive carcinomas, should provide a provisional estimate of the degree of differentiation and the growth pattern. In the oral cavity, the depth of invasion or tissues involved (mucosa, muscle) may guide the extent of surgery.

Resection specimens provide sufficient tissue to describe the full range of prognostic information^2^^,^^11^ (Box I); the basis in evidence for this information is provided in guidelines published by the Royal College of Pathologists and varies between anatomical sites.

BOX I **PROGNOSTIC INFORMATION DERIVED FROM PRIMARY CARCINOMAS**

Site and subsite

Histological type of carcinoma

Grade of differentiation

Growth pattern

Maximum diameter

Maximum depth of invasion

Invasion of lymphatic or blood vessels

Invasion of the peri-neural space of nerve trunks

Invasion of bone or cartilage

Distance of carcinoma from resection margins

### Dysplasia and intra-epithelial neoplasia

Squamous cell carcinomas are the result of a combination of genetic mutations, some of which are manifest in precursor lesions by atypia of the epithelial cells collectively referred to as dysplasia or intra-epithelial neoplasia. Severe cytological atypia is associated with a high risk of progression to carcinoma and, in resection specimens, its presence at resection margins may predict local recurrence. The various, commonly used, grading systems are summarised in Table I and, although different criteria are used, each seeks to place a particular abnormality in a continuous spectrum of appearances from mild to severe atypia. There is no UK consensus^12^ on which grading system should be recommended, although a majority of pathologists probably use the WHO dysplasia system but regard severe dysplasia and in situ carcinoma as indistinguishable. A proposed consensus system for laryngeal lesions based on the Ljubljana classification^13^ is gaining recognition, but its translation to UK practice is limited. Management decisions should take account of the microscopic severity of the lesion and its clinically assessed extent.

**Table I:** Grading systems for precursor lesions of squamous epithelial malignancies

WHO Classification 2005	Squamous intraepithelial neoplasia (SIN)	Ljubljana classification; squamous intraepithelial lesions (SILs)
Squamous cell hyperplasia		Simple hyperplasia
Mild dysplasia	SIN 1	Basal/parabasal cell hyperplasia
Moderate dysplasia	SIN 2	Low grade SIL
Severe dysplasia	SIN 3	High grade SIL
Carcinoma in situ	SIN3	Carcinoma in situ

Note: The categories in the different systems are not strictly comparable as different morphological and architectural criteria are used

### Other mucosal malignancies

#### Adenocarcinomas

This may be of surface or salivary type. Those derived from surface epithelium of the nose and sinuses may resemble intestinal carcinomas and have a relatively poor prognosis compared with other low grade adenocarcinomas.

#### Sinonasal undifferentiated (anaplastic) carcinoma

This is a rare, clinically aggressive neoplasm composed of cells that are undifferentiated on routine stains but which show varying degrees of neuroendocrine differentiation on immunocytochemistry. These carcinomas often result in bone destruction and extension into the orbit or cranial cavity and have a poor prognosis despite aggressive surgery and chemoradiotherapy.

#### Olfactory neuroblastoma (esthesioneuroblastoma)

This presents as a polypoid mass high in the nasal cavity. The histological features are characteristic and immunocytochemistry is positive for neuroendocrine markers. Morphological grading systems are of limited prognostic value. Despite spread to regional nodes and more distant sites, prognosis is good with a 78 per cent five-year survival after surgery and RT.

#### Malignant melanoma

This most often arises in the nasal cavities and less often in the sinuses, presenting in adults over 50 years as polypoid, friable haemorrhagic masses. Histologically there is a wide range of appearances with very variable melanin production (30 per cent are amelanotic). Survival is poor with death due to widespread metastasis and/or extensive local recurrence.

#### Lymphomas

This may present as primary mucosal malignancies in the sinonasal tract and tonsils. Almost all are non-Hodgkin's lymphomas with natural killer/ T-cell lymphomas mainly affecting the sinonasal tract and B-cell lymphomas arising in the tonsils.

#### Nasopharyngeal carcinoma

This includes keratinising SCCs and non-keratinising differentiated and undifferentiated carcinomas and is usually related to Epstein–Barr virus infection. The synonym of ‘lymphoepithelioma’ should not be used. Keratinising carcinomas are more radioresistant than non-keratinising and undifferentiated carcinomas.

## Diagnosis and management of neck lumps

### Fine needle aspiration

Fine needle aspiration (FNA) of tissue by a well-trained operator is an essential part of the diagnostic assessment of patients with neck or thyroid lumps and as part of staging procedures for patients with the known head and neck cancer.^14^^,^^15^ High-quality preparations are essential for an effective service. Either rapidly air-dried slides or needle washings into preservative solution may be required depending on the clinical circumstance. The cytological diagnosis of metastatic SCC in cervical nodes is usually straightforward, but cystic metastases can be difficult to distinguish from benign cystic lesions containing squamous cells such as branchial cleft cysts; a high degree of clinical suspicion for malignancy is required in older patients with cystic lesions containing squamous cells. Haemorrhage into cystic neck nodes may conceal underlying malignancy, particularly metastatic papillary carcinoma from the thyroid. Multidisciplinary correlation of findings is of fundamental importance.

FNA cytology is the method of choice for monitoring patients known to have lymphoma as cytology can document disease recurrence and can indicate transformation from low to high grade disease. The primary diagnosis of lymphoma can be made from FNA specimens if the laboratory repertoire includes molecular techniques and flow cytometry. FNA cytology is an effective method to triage patients into those in whom significant disease can be excluded, those in whom a definitive diagnosis of benign disease or metastatic malignancy can be made, and those with possible lymphoma who need lymph node biopsy. Where malignancy is identified, additional immunocytochemical and molecular testing for planning management is possible with appropriate specimen collection procedures.

### Neck dissections

The presence or absence of nodal metastasis is a key component of tumour–node–metastasis (TNM)^16^ staging and determines further management. The pathological assessment of nodes in resection specimens verifies pre-operative imaging studies and identifies small volume nodal disease that is beyond the resolution of current imaging techniques.

The terminology of possible nodal involvement by carcinoma includes:

• Isolated tumour cells (ITCs) – collections of cells <0.2 mm diameter
• Micrometastasis – tumour deposits 0.2–2 mm in diameter
• Conventional metastasis – a tumour deposit more than 2 mm diameter
• Extracapsular spread – carcinoma extending through a breach in the capsule from a lymph node into surrounding connective tissue.

For TNM staging, the presence of ITCs is classified as pN0 as their significance is unknown. Micrometastases are recorded as pN1(mi), pN2b(mi) or pN2C according to their extent in multiple nodes. Core pathological data for nodal metastases are shown in Box II.

BOX II **PROGNOSTIC INFORMATION DERIVED FROM LYMPH NODE EXCISIONS**

Number of positive nodes

Sites of positive nodes

Size of largest metastasis

Presence or absence of extracapsular spread

## Salivary neoplasms

Most tumours arising in the major or minor salivary glands are benign (although the proportions vary from site to site), but pre-operative suspicion of malignancy may be raised on clinical examination, from imaging studies or from pre-operative FNA cytology. All tumours of the major salivary glands should have pre-operative FNA cytology to guide treatment, which can usually accurately diagnose pleomorphic salivary adenoma and Warthin's tumour with confidence, differentiate benign neoplasms from malignant in 81–98 per cent of cases, but which is less good at establishing a specific type of carcinoma. The main categories of salivary carcinoma are well defined, but these tumours have many morphological variants and precise histological diagnosis often requires a specialist opinion. Many salivary neoplasms have characteristic genetic translocations^17^ which aid diagnosis and may lead to targeted therapeutics. The core pathological data from salivary resections for neoplasia are shown in Box III.

BOX III **PROGNOSTIC INFORMATION DERIVED FROM SALIVARY GLAND RESECTIONS**

The histological type of neoplasm (according to the WHO Classification)

The grade of malignancy (see text)

The distance to the resection margins

The presence or absence of peri-neural or vascular invasion

The presence or absence of lymph node involvement

Grading of the degree of malignancy is prognostically useful for some salivary carcinomas. Grading of mucoepidermoid carcinomas relates to metastatic potential and survival, whichever grading system is used. Acinic cell carcinomas are usually circumscribed but incompletely encapsulated; grading on the basis of cytological features is not generally useful, except for rare tumours showing dedifferentiation. Assessment of Ki-67 (MIB1) labelling is of prognostic value, and acinic cell carcinomas with indices of >5 per cent behaving more aggressively. The growth pattern of adenoid cystic carcinoma is related to metastatic potential, with 0–4 per cent of cribriform, hyaline and tubular carcinomas, and 33 per cent of solid (basaloid) carcinomas metastasising to local lymph nodes. Distant metastasis is more common in solid tumours. Salivary duct carcinoma is a high-grade malignancy morphologically resembling ductal carcinoma of the breast. About 70 per cent express androgen receptors and 15 per cent express HER-2 (human epithelial growth factor receptor 2); features which may influence therapy. Carcinomas arising in pleomorphic adenomas may be of any or mixed histological type; the extent of invasion is prognostically useful as invasion more than 5–6 mm from the capsule of the residual adenoma is associated with a high risk of local recurrence and distant metastasis. Non-invasive or minimally invasive carcinomas ex pleomorphic adenoma are true malignancies, but have a very low rate of disease progression.

## Thyroid cancers

Most lesions will have had FNA before surgery. Immediate assessment of the adequacy of aspirates may be helpful. The descriptive cytology report informs clinical decisions on management and should incorporate a categorical summary^3^^,^^18^ (Table II).

**Table II:** Categorisation of thyroid FNAs with likelihood of malignancy (LOM) (RCPath and BSCC guidelines)

Thy 1	Non-diagnostic for cytological diagnosis	LOM 0–10%
Thy 1c	Non-diagnostic for cytological diagnosis – cystic lesion	
Thy 2	Non-neoplastic	LOM 0–3%
Thy 2c	Non-neoplastic, cystic lesion	
Thy 3a	Neoplasm possible – atypia/non-diagnostic	LOM 5–15%
Thy 3f	Neoplasm possible, suggesting follicular neoplasm	LOM 15–30%
Thy 4	Suspicious of malignancy	LOM 60–75%
Thy 5	Malignant	LOM 97–100%

For all malignant thyroid tumours, the national dataset for histopathology reports^3^ defines core data items of prognosis importance that will allow TNM staging^16^ (Box IV). Some histological variants of thyroid carcinomas have prognostic importance. For diagnostic purposes, oncocytic (Hürthle cell) follicular tumours are regarded as a variant of follicular tumours and the criteria for malignancy are the same. The presence of any poorly differentiated or anaplastic component affects prognosis.^3^

BOX IV **PROGNOSTIC DATA FROM THYROID RESECTION SPECIMENS**

Histological type of malignancy

Whether carcinoma is unifocal or multifocal

Maximum dimension of carcinoma (largest if multifocal)

Closest distance to surgical resection margin (R status)

Extension into extrathyroidal tissues (macroscopic or microscopic)

Presence of lymphatic or vascular invasion

Site and number of lymph nodes sampled and those involved

### Papillary carcinoma

A single papillary microcarcinoma (≤10 mm diameter) discovered incidentally in a resection performed for another disease is not thought to have a significant risk of recurrence or metastasis. Some microcarcinomas are potentially more aggressive including those with multifocal disease, extrathyroid extension and lymphatic invasion.

Tall cell and columnar variants of papillary carcinoma may be more aggressive, while the outcome of the diffuse sclerosing variant is a matter of debate.

Diagnosis of the follicular variant of papillary carcinoma (FVPC) may be difficult and require specialist opinion. The non-encapsulated invasive FVPC has a metastatic potential similar to that of classical papillary carcinoma, while encapsulated FVPC has metastatic potential related to the number of foci of vascular invasion.

### Follicular carcinoma

A follicular neoplasm is defined as carcinoma on the basis of capsular and/or vascular invasion. Minimally invasive follicular carcinomas show only focal microscopic vascular and/or capsular invasion. Tumours showing only capsular invasion have a minimal risk of metastasis. The risk of metastasis increases with vascular invasion, but no significance is attached to the number of foci of vascular invasion. Widely invasive follicular carcinoma shows obvious gross invasion or extensive microscopic infiltration of thyroid parenchyma, vessels or extrathyroidal tissues. The number of foci of vascular invasion should be described but is not prognostically significant.

### Medullary carcinoma

The diagnosis should be confirmed by calcitonin immunoreactivity, although some poorly differentiated carcinomas only express carcinoembryonic antigen (CEA). Although there are variations in the cellular pattern and presence of amyloid these are unimportant prognostically compared with the tumour stage and completeness of excision. In the syndromes of multiple endocrine neoplasia type 2 and familial medullary thyroid carcinoma, medullary carcinoma is often multifocal and preceded and/or accompanied by C-cell hyperplasia. Genetic testing for RET mutations will detect familial syndromes.

### Poorly differentiated carcinoma

This group is defined as follicular or papillary carcinoma with necrosis and/or a mitotic count of five or more in ten high-power microscopic fields. The growth pattern may be insular, trabecular or solid. Poorly differentiated carcinomas have a poorer prognosis than differentiated carcinomas with variable response to radio-iodine treatment.

### Undifferentiated/anaplastic carcinoma

Anaplastic carcinoma is diagnosed where a follicular or papillary carcinoma shows even a minor undifferentiated (anaplastic) component. Most undifferentiated tumours will be diagnosed by FNA cytology, core or open biopsy and will not have a surgical resection. The report should describe how immunocytochemistry has been used to exclude other poorly differentiated malignancies, especially lymphoma.

### Lymphoma

The diagnosis of thyroid lymphoma is usually made on core or open biopsy rather than resection specimens and may require extensive immunocytochemical and molecular testing. It is important to distinguish between primary thyroid lymphoma and involvement of the thyroid by lymphoma as part of a wider disease.

**Recommendations**

• Accurate diagnosis of the type of malignancy is a key component of effective management (R).
• Surgeons and oncologists should understand the scope and limitations of cellular pathology in order to inform multidisciplinary discussions (R)
• A clinically suspected diagnosis of malignancy should be confirmed by biopsy or cytology before operation (R)
• Cytopathological diagnoses should be discussed with surgeons and radiologists to maximise the information gained from each modality of investigation (R)
• Pathological investigations are the basis for accurate cancer staging and stratification of clinical outcomes (R)
